# Genetic stability of foot-and-mouth disease virus during long-term infections in natural hosts

**DOI:** 10.1371/journal.pone.0190977

**Published:** 2018-02-01

**Authors:** Lisbeth Ramirez-Carvajal, Steven J. Pauszek, Zaheer Ahmed, Umer Farooq, Khalid Naeem, Reed S. Shabman, Timothy B. Stockwell, Luis L. Rodriguez

**Affiliations:** 1 Foreign Animal Disease Research Unit, Agricultural Research Service, Plum Island Animal Disease Center, New York, United States of America; 2 Oak Ridge Institute for Science and Education (ORISE), Oak Ridge, Tennessee, United States of America; 3 Foreign Animal Disease Diagnostic Laboratory, Animal Plant Health Inspection Service (APHIS), Plum Island Animal Disease Center, New York, United States of America; 4 Animal Health Program, National Agricultural Research Center, Islamabad, Pakistan; 5 J. Craig Venter Institute, Rockville, Maryland, United States of America; Oklahoma State University, UNITED STATES

## Abstract

Foot-and-mouth disease (FMD) is a severe infection caused by a picornavirus that affects livestock and wildlife. Persistence in ruminants is a well-documented feature of Foot-and-mouth disease virus (FMDV) pathogenesis and a major concern for disease control. Persistently infected animals harbor virus for extended periods, providing a unique opportunity to study within-host virus evolution. This study investigated the genetic dynamics of FMDV during persistent infections of naturally infected Asian buffalo. Using next-generation sequencing (NGS) we obtained 21 near complete FMDV genome sequences from 12 sub-clinically infected buffalo over a period of one year. Four animals yielded only one virus isolate and one yielded two isolates of different serotype suggesting a serial infection. Seven persistently infected animals yielded more than one virus of the same serotype showing a long-term intra-host viral genetic divergence at the consensus level of less than 2.5%. Quasi-species analysis showed few nucleotide variants and non-synonymous substitutions of progeny virus despite intra-host persistence of up to 152 days. Phylogenetic analyses of serotype Asia-1 VP1 sequences clustered all viruses from persistent animals with Group VII viruses circulating in Pakistan in 2011, but distinct from those circulating on 2008–2009. Furthermore, signature amino acid (aa) substitutions were found in the antigenically relevant VP1 of persistent viruses compared with viruses from 2008–2009. Intra-host purifying selective pressure was observed, with few codons in structural proteins undergoing positive selection. However, FMD persistent viruses did not show a clear pattern of antigenic selection. Our findings provide insight into the evolutionary dynamics of FMDV populations within naturally occurring subclinical and persistent infections that may have implications to vaccination strategies in the region.

## Introduction

Foot-and-mouth disease virus (FMDV) causes a severe and highly contagious acute vesicular illness that affects livestock and wildlife. The wide genetic and antigenic variation of this virus is evidenced by the existence of 7 serotypes (A, O, Asia-1, C, SAT1, SAT2, SAT3) and multiple subtypes [1,2]. Although vaccines are available, no protection across serotypes is observed. Thousands of animals in endemic regions are still affected, representing a major sanitary constraint to trade of animal products [3].

FMDV is a single-stranded positive-sense RNA picornavirus with an ~8.3 Kb genome that encodes an ~7Kb single open reading frame (ORF) flanked by the 5′ and 3′ untranslated regions (UTRs) [4]. The polyprotein is co- and post-translationally processed through cellular and viral proteases [4]. The Leader protease (Lpro) is located at the 5′-end of the ORF, the P1 region comprises the virion structural proteins VP1 [1D], VP2 [1B], VP3 [1C], and VP4 [1A] [2,4], the P2 region encompasses non-structural proteins processed into three mature polypeptides, 2A, 2B, 2C [4], and the P3 region includes the 3A, 3B, 3Cpro and 3D (polymerase) [2,4].

Phylogenetic and molecular epidemiology studies, mostly based on VP1 sequencing of virus from clinical lesions, have been valuable tools to trace FMD spread during outbreaks [5]. Yet, complete genome sequencing provides a better tool to trace within and between herd transmission [6, 7]. However, only a few studies report findings from complete genomes of viruses causing subclinical infections.

FMD is endemic in the South Asian region, and regular outbreaks are reported in the Indo-Pakistan subcontinent [8]. Official reports of diagnoses and mortalities caused by FMD in Pakistan date back to 1937 [9] and four serotypes (A, O, C and Asia-1) have been reported in this territory since [10]. From 1952 to 2007, a 62% prevalence of FMDV across clinically suspected cases for FMD epidemics was reported [10]. Serotype O was the most prevalent followed by serotype Asia-1, and A. Serotype C was last detected in 1995 [10]. More recent studies have shown that serotypes O [11], [12], A [13], and Asia-1 [14] were responsible for most of the outbreaks during the past 10 years [14, 15, 16]. Epidemiological factors that could contribute to nucleotide (nt) and amino acid aa) variation of viruses circulating in the region have also being studied [11]

Besides acute vesicular disease, FMDV also causes subclinical and persistent infection in the pharyngeal tissues of ruminants, yet the mechanisms of FMDV persistence are not fully understood. Persistently infected ruminants (i.e. carriers) intermittently shed virus for long periods (months to years) and are a perceived risk of virus transmission [17, 18]. However, the role of persistent infections in transmission is unclear [19, 18]. Natural [20] and experimental [21] data supports FMDV transmission from persistently infected African buffalo (*Syncerus caffer*) to cattle, but more recent studies failed to show FMDV transmission from African buffalo to cattle despite prolonged direct contact and immunosuppressive treatment of susceptible cattle [19].

Although persistent infections have been previously reported in Asian buffalo [22], little information is available on the role of carrier Asian buffalo in FMDV transmission in endemic regions particularly, since unlike their African counterparts, the majority of Asian buffalo are domesticated and play an important role for meat and milk production in large parts of Asia. Furthermore, little is known about the host-virus interactions and selective pressures exerted during viral replication throughout persistence.

Here we analyzed sequences of FMDV isolates obtained from oropharyngeal fluids (probang) collected from subclinically infected and carrier Asian buffalo kept under peri-urban dairy farming conditions in Pakistan. We assembled 21 near complete FMDV genomes using next generation sequencing (NGS) strategies [23]. Little genetic divergence was observed during intra-host persistence evidenced by close relatedness at the consensus level and discovery of few minor variants with a frequency >25%. In addition, we identified signature amino acid substitutions and conserved antigenic regions of persistent viruses compared to other sequences available in public databases. Phylogenetic analysis of Asia-1 VP1 sequences of this study were classified within group VII, along with other Pakistani isolates derived from acute infections collected in 2011. No evidence of inter- or intra-serotypic recombination was found. This study afforded a unique opportunity to study viral evolution and diversity during persistence in an endemic region. Studies of FMDV genome sequence diversity, population dynamics, and phylogeny may contribute to understanding the role of persistently infected animals in FMDV ecology.

## Materials and methods

### Sample source

Viruses analyzed in this study were derived from oro-pharyngeal fluid (probang) samples collected from Asian water buffalos (*Bubalus bubalis*) in 30 dairy farms near Islamabad, Pakistan. Samples from this study were collected over a one-year period as part of a collaborative study to understand subclinical FMD infections in buffalo [24] and to study outbreaks occurring in Pakistan between 2008–2012 [25]. The geographical distribution of herds from the collaborative study (2008–2012) was been recently published [25]. There were no FMD outbreaks reported in any of the 30 farms during the study period [24].

The subset of samples for this study included viruses from 12 FMDV asymptomatic infected buffalos quarterly sampled between December 2011 and December 2012, (Table 1). Samples were shipped to the Plum Island Animal Disease Center (PIADC) for further processing. Of the 4 samples collected per animal at different times of the year, not all resulted positive for PCR and viral isolation (VI). Of the 12 animals, 7 were confirmed persistently infected with isolation of the same virus serotype more than 28 days apart [18] (Table 1). Four other animals were subclinically infected but we cannot confirm persistence since virus was isolated only one time (Table 1). Finally, one animal was serially infected with two different viral serotypes isolated on two separate quarterly samplings (Table 1). Only samples positive for PCR and VI were used for genome sequencing in this study. Genomes derived from the same animal at different time points were labeled with the corresponding animal identification (ID) followed by chronological order of the sampling time, denoted as 1, 2, 3, or 4, corresponding to the first, second, third, and fourth sampling point, respectively (Table 1). In this study, an ancestral sequence is defined as the viral consensus sequence collected at the earliest time point from the same persistently infected animal.

**Table 1:** Sample information and reads statistics of FMDV isolated from natural persistently infected buffalos.

Animal ID	Sample ID	BioSample accession*	Collection date	Days between sampling	Serotype	Mean read length	FMDV mapped read count	Reads without duplicates	Coverage of FMDV CDS (%)	Finishing by Sanger	Average coverage level	Reference genome accession #
8^a^	8–1	SAMN05437881	28-Dec-11	0	Asia1	178	4412	3211	98	Yes	61	KM268898
	8–3	SAMN05437882	17-May-12	141	A	166	63378	26353	99	Yes	589	JN006722
137^b^	137–1	SAMN05437883	31-Jan-12	0	Asia1	196	28471	16002	99	Yes	358	KM268898
161^c^	161–1	SAMN05437884	2-Feb-12	0	O	171	18312	11950	100	No	272	HQ268524
	161–2	SAMN05437885	29-Mar-12	56	O	172	71333	19984	100	No	354	HQ268524
178^c^	178–2	SAMN05437886	03-Apr-12	0	Asia1	173	8767	5560	99	Yes	112	KM268898
	178–3	SAMN05437887	09-Jul-12	97	Asia1	210	18267	11009	100	No	298	KM268898
184^c^	184–1	SAMN05437888	08-Feb-12	0	Asia1	169	2168	1652	98	Yes	35	KM268898
	184–2	SAMN05437889	03-Apr-12	55	Asia1	178	3286	2369	99	Yes	37	KM268898
185^c^	185–1	SAMN05437890	08-Feb-12	0	Asia1	195	40035	13584	100	No	353	KM268898
	185–3	SAMN05437891	09-Jul-12	152	Asia1	164	11241	6386	100	No	134	KM268898
189^b^	189–3	SAMN05437892	11-Jul-12	0	Asia1	209	4488	3026	99	Yes	70	JN006719
192^c^	192–2	SAMN05437893	04-Apr-12	0	Asia1	164	36293	14003	100	No	297	KM268898
	192–3	SAMN05437894	11-Jul-12	98	Asia1	156	3801	2567	100	No	60	KM268898
	192–4	SAMN05437895	22-Nov-12	232	Asia1	180	835	774	94	Yes	16	KM268898
230^b^	230–4	SAMN05437896	03-Dec-12	0	Asia1	144	7554	4938	100	No	110	KM268898
231^b^	231–4	SAMN05437897	3-Dec-12	0	Asia1	198	12651	8444	100	No	203	JN006719
254^c^	254–1	SAMN05437898	23-Feb-12	0	Asia1	169	25897	12845	100	No	308	KM268898
	254–4	SAMN05437899	12-Dec-12	293	Asia1	186	10966	6014	97	Yes	76	KM268898
277^c^	277–1	SAMN05437900	29-Feb-12	0	Asia1	182	16383	8913	100	No	155	KM268898
	277–4	SAMN05437901	11-Dec-12	286	Asia1	163	1670	1180	95	Yes	24	KM268898

*SRA accession: SRP079712.

^a^serially infected animal;

^b^ subclinically infected animal;

^c^ confirmed persistently infected animal

### Virus isolation

The amount of virus in the probangs was not enough to obtain viral RNA appropriate for direct NGS library preparation. For this reason, viral isolation was carried out in LFBK αVβ6 cells as previously described [26] and passage 1 supernatants were used for library preparation. Briefly, probang samples were treated with trichlorotrifluoroethane (TTE) and clarified by centrifugation. Semi confluent cells seeded in a T25 flask were rinsed with serum free media and inoculated with 150 μl of the clarified probang sample. After 1 h of adsorption at 37°C on a rocker plate, 5 ml of cell maintenance media with 1% serum was added to each flask and flasks were incubated at 37°C. Supernatants from samples showing cytopathic effect (CPE) within 24–48 h post inoculation were collected and stored at −70°C for further testing.

### FMDV RNA detection

RNA was isolated using a KingFisher magnetic particle processor (Thermo scientific, Hudson, NH) according to the manufacturer’s instructions. Fifty microliters of cell culture supernatant from each viral isolate was used for RNA isolation. FMDV specific RNA was quantified by rRT-PCR from cell culture supernatants on a 7500 Thermocycler (Applied Biosystems, Life Technologies, Carlsbad, CA) following a protocol previously described [27]. Samples were considered positive when threshold cycles (C_T_) were <40.

### Sequence-Independent, Single-Primer Amplification (SISPA) library preparation and sequencing

Genomic libraries were generated as previously described [23] using a high-throughput 96-well format and unique barcoding of each sample. The use of barcodes (indexes) allowed pooling of the samples before any platform specific protocol (Illumina MiSeq or Ion torrent) was followed. Briefly, random hexamer oligonucleotides coupled with unique barcodes were used for first strand cDNA synthesis (Life Technologies, Carlsbad, CA). A Klenow fragment DNA polymerase was used for second strand DNA synthesis (New England Biolabs, Ipswich, MA). PCR amplification was carried out using primers matching the 5’ barcode used in reverse transcription and resulted in barcoded PCR products ranging from ~300–1000 bp in size. Amplified PCR products were treated with an RNAse cocktail and column purified (Qiagen Valencia, CA). Final SISPA products were normalized, pooled and either Illumina or Ion torrent adapters were ligated onto the pooled amplicons. For Illumina Miseq sequencing, the New England Biolabs (NEB) end prep and ligation modules (E7546S, E7595S) and Bio Scientific barcoded adapters (514113) were used. Libraries were purified, size-selected, visualized, and quantified. Approximately 9 pM of a final library pool was loaded on a MiSeq 2x300 cycle reagent cartridge for sequencing. For Ion Torrent PGM sequencing, blunt ends were created using the end repair enzyme from the Ion plus Fragment Library kit (Life Technologies, Carlsbad, CA) and fragments were ligated to Ion Torrent PGM-compatible barcode adapters. Libraries were purified, size-selected, visualized, quantified and pooled. The resulting multiplexed library was loaded on an Ion OneTouch 2 instrument (Life Technologies) to perform emulsion PCR on Ion Sphere particles using the Ion PGM Template OT2 400 kit. The ion sphere particles were deposited in the Ion 318 chip v2 (revision 2.0, Life Technologies) for sequencing.

### Post-sequencing genomic analyses and finishing

Post-sequencing genomic analyses were conducted using CLCbio and CLC Genomics Workbench version 8.5.1 (CLC Bio, Qiagen). Briefly, adaptor contamination removal and quality trimming (Phred score ≥ 20) was conducted. Reads were demultiplexed based on specific SISPA barcodes using J. Craig Venter Institute (JCVI) custom software. Duplicate reads were omitted. Reads tagged with different barcodes but belonging to the same sample were combined for mapping. Reads were *de novo* assembled and contigs over 500nt in length were BLASTed to identify appropriate reference genome sequences available from public sequence databases. After selecting the most appropriate reference genome, reads were mapped and non-specific matches were ignored. Partial or complete FMDV genome sequences were obtained. When required, ORFs were finished using Sanger sequencing. For Sanger sequencing, RT-PCR products were generated using SuperScript^®^III One-Step RT-PCR System with Platinum^®^ Taq High Fidelity (Life Technologies, Carlsbad, CA), column purified, and sequenced using the di-deoxy termination method (Big dye terminator; (Life Technologies, Carlsbad, CA). Chromatograms were analyzed using Sequencher^®^ v5.1 (GeneCodes) and a consensus sequence generated by NGS was updated to include the Sanger derived sequence.

### Virus consensus sequences

ORF consensus sequences were generated after mapping assembly of the reads to the closest reference genome available in public databases. Annotation of the reference genome was based on predictive information available at Virus pathogen resource (ViPR) (www.viprbrc.org). For SNV analysis of samples 161–2, 178–3, and 185–3, the consensus sequence of the ancestral isolate functioned as the reference genome to map reads. Nucleotide data conflicts were resolved by choosing the base with the highest additive quality score. Genome finishing was conducted by Sanger sequencing as described above.

### Analyses of nucleotide variants and non-synonymous amino acid substitutions

For samples 161–2, 178–3 and 185–3, SNVs were determined using the consensus sequence of the ancestral sample as the reference genome for mapping. Regions with low central base quality score were excluded from the analysis. A probabilistic (α = 0.05, minimum variant count = 10, minimum frequency = 0.25) and quality-based (central quality ≥20) test built in CLC Genomics Workbench version 8.5.1 was used to minimize false positive SNP calls caused by sequence-specific errors. For each selected pair of isolates, the progeny virus was the same serotype as the ancestral virus, the mean coverage was ≥ 100X, and variants were considered only in genomic regions with a depth of coverage ≥ 200X.

SNV with the lowest frequency were considered minor variants (with frequencies between 25 to 49%). Minor variants (minimum 25% frequency) or dominant variants (occurring with >90% frequency) are reported compared to the reference genome.

The amino acid sequences, synonymous and non-synonymous amino acid substitutions were deduced using CLC Genomics Workbench version 8.5.1.

### Phylogenetic analysis

VP1 sequences or consensus ORFs of Asia-1 viruses were aligned using ClustalW [28] implemented in CLC Genomics Workbench version 8.5.1. Additional sequences available from NCBI were included in the phylogenetic analysis and accession numbers are denoted in each figure. The evolutionary parameters were estimated using MEGA 7 [29] as specified in each figure legend. The evolutionary history was inferred by the Maximum Likelihood (ML) method based on ORF (GTR model) or VP1 (HKY model) sequences of Asia-1 isolates. The bootstrap consensus tree inferred from 1000 replicates is shown. To infer the phylogenetic relationship of Asia-1 VP-1 sequences from this study, with sequences from Turkey and additional sequences, a Bayesian analysis was performed using MrBayes (v3.2) [30] with the following settings: the evolutionary model was set to GTR substitution model (nst = 6) with gamma-distributed rate variation across sites and a proportion of invariable sites (rates = invgamma). The Markov chain Monte Carlo search was run with 4 chains for 1000000 generations, with trees being sampled every 1000 generations (25% samples from the cold chain were discarded as "burnin"). Additional representative sequences from groups I-VIII of Asia-1 serotype were selected based on previous reports [14, 16, 31, 32].

### Estimation of selective pressure

A pairwise intra-host comparison between non-synonymous mutations (Ka), and synonymous mutations (Ks) for each sequence was conducted using the KaKs Calculator [33]. To detect selective pressure on individual sites of codon alignments, Asia-1 sequences from this study were aligned and compared using the selective pressure detection tool Datamonkey [34] at default parameters. Comparison of results from SLAC/FEL/REL [35] and Mixed Effects Model of Episodic Diversifying Selection (MEME) [36] methods was carried out. FUBAR Approach to Directional Evolution (FADE) analysis was conducted on the equivalent amino acid sequences implemented in Datamonkey platform [37].

### Mapping of antigenic sites

Structural or non-structural FMDV amino acid motifs previously reported as antigenic (n = 39) [38] were mapped on the deduced amino acid sequences of samples from this study (n = 21) using the motif search tool in CLC Genomics Workbench version 8.5.1. Conserved antigenic sites of viral structural proteins [38] were identified for Asia-1 isolates. Epitopes located in structural proteins were also searched in a subset of Asia-1 protein sequences available from NCBI database (Accession numbers: AMB19068, AGI05089, ALF36833, AFE84733, AIT55229, ABF74751, AGJ03147, AEQ49431, AEQ49430, AEB00690, AEO22161, ADV52249, ACP44144, ADU56664, ADX97245, ADB28902, ADC92544, ADC92543, ABI93996, ABI93995, ABI93994, ABI93993, ABI93992, ABI93991, ABI93990, ABI93989, ABI93988, ABI93987, ABI93986, ABI93985, ABI93984, ABI93983, ABI93982, ABI93981, ABI93980, ABI93979, ABI93978, ABI93977, ABI93976, AAT01743, AAT01742, AAT01741, AAT01740, AAT01739, AAT01738, AAP60035, AAU00942, AAU00941, AAQ90285).

### Recombination analysis

Similarity plots were carried out with Simplot [39] for samples 8–1 and 8–3 including strains Asia-1 TURK 2013 (Accession number KM268898), Asia-1 SIN/PAK/L5 2008 (Accesion number JN006719), and A SIN/PAK/L4 2008 (Accession number JN006722) in the analyses. The genetic algorithm for recombination detection (GARD) [40] was used to analyze recombination among isolates from this study.

### Sequence data

The sequencing reads mapped to the selected reference genome were submitted to NCBI’s Sequence Read Archive and can be found under the Submission ID: SRP079712.

## Results

### High-throughput sequencing of FMDV genomes

High-throughput sequencing of FMDV genomes allows for in-depth analysis of FMDV diversity. Twenty one near complete FMDV genomes were recovered in this study (Table 1). Serotype, determined by blasting *de novo* assembled contigs to public databases, was confirmed by partial sequencing of VP1 by Sanger method (not shown). Using both Sanger and NGS, 18 samples were classified as Asia-1, two samples corresponded to serotype O, and one sample as serotype A. The read count that mapped to FMDV genomes and other statistics of each assembly varied considerably across the samples and are shown in Table 1.

#### Determination of Inter- and intra-host genomic variability in persistent infections

The Asia-1 samples from this study shared >98% nt identity at the consensus level (Table 2), except for sample 254–1. At the consensus level, the number of divergent nucleotides from viral isolates of the same animal ranged from 0 to 978 nt.

**Table 2:** Comparison of the number of nucleotide differences (from 0 to 1000 nt) or percentage of identity (from 85 to 100%) across isolates from the study.

Animal ID	8–1	137–1	178–2	178–3	184–1	184–2	185–1	185–3	189–3	192–2	192–3	192–4	230–4	231–1	254–1	254–4	277–1	277–4	8–3	161–2	161–1
8–1	-	15	18	11	24	17	12	15	22	19	20	23	56	52	132	52	31	59	978	919	919
137–1	99.79	-	23	16	29	22	17	20	27	24	25	28	61	57	133	57	36	64	978	919	919
178–2	99.74	99.67	-	7	20	13	8	11	30	27	28	31	64	60	138	60	39	67	984	924	924
178–3	99.84	99.77	99.90	-	13	6	1	4	23	20	21	24	57	53	131	53	32	60	980	922	922
184–1	99.66	99.59	99.71	99.81	-	16	14	17	36	33	34	37	70	66	144	66	45	73	992	934	934
184–2	99.76	99.69	99.81	99.91	99.77	-	7	10	29	26	27	30	63	59	137	59	38	66	986	926	926
185–1	99.83	99.76	99.89	99.99	99.80	99.90	-	3	24	19	20	23	58	54	130	54	33	61	979	921	921
185–3	99.79	99.71	99.84	99.94	99.76	99.86	99.96	-	27	22	23	26	61	57	133	57	36	64	981	924	924
189–3	99.69	99.61	99.57	99.67	99.48	99.59	99.66	99.61	-	9	10	13	66	62	142	62	41	71	984	927	927
192–2	99.73	99.66	99.61	99.71	99.53	99.63	99.73	99.69	99.87	-	3	6	63	59	139	59	38	68	979	923	923
192–3	99.71	99.64	99.60	99.70	99.51	99.61	99.71	99.67	99.86	99.96	-	7	64	60	140	60	39	69	980	924	924
192–4	99.67	99.60	99.56	99.66	99.47	99.57	99.67	99.63	99.81	99.91	99.90	-	67	63	143	63	42	72	981	925	925
230–4	99.20	99.93	99.08	99.18	99.00	99.10	99.17	99.13	99.06	99.10	99.08	99.04	-	10	171	6	77	21	994	938	938
231–1	99.26	99.18	99.14	99.24	99.06	99.16	99.23	99.18	99.11	99.16	99.14	99.10	99.86	-	163	4	73	21	988	933	933
254–1	98.11	98.10	98.03	98.13	97.94	98.04	98.14	98.10	97.97	98.01	98.00	97.95	97.55	97.67	-	167	149	173	1005	942	942
254–4	99.26	99.18	99.14	99.24	99.06	99.16	99.23	99.18	99.11	99.16	99.14	99.10	99.91	99.94	97.61	-	73	17	992	934	934
277–1	99.56	99.48	99.44	99.54	99.36	99.46	99.53	99.48	99.41	99.46	99.44	99.40	98.90	98.96	97.87	98.96	-	80	989	934	934
277–4	99.16	99.08	99.04	99.14	98.96	99.06	99.13	99.08	98.98	99.03	99.01	98.97	99.70	99.70	97.53	99.76	98.86	-	1000	940	940
8–3	86.04	86.04	85.96	86.01	85.84	85.93	86.03	86.00	85.96	86.03	86.01	86.00	85.81	85.90	85.66	85.84	85.89	85.73	-	946	946
161–2	86.88	86.88	86.81	86.84	86.67	86.78	86.85	86.81	86.77	86.82	86.81	86.80	86.61	86.68	86.55	86.67	86.67	86.58	86.50	-	0
161–1	86.88	86.88	86.81	86.84	86.67	86.78	86.85	86.81	86.77	86.82	86.81	86.80	86.61	86.68	86.55	86.67	86.67	86.58	86.50	100	-

Highlighted in gray: Samples from serotypes A or O as compared to Asia-1 isolates

As viral quasispecies are subjected to competition and selection within a mutation-prone environment [41], we sought to determine genetic variation of viruses during persistent infections. Three pairs of viral isolates derived from an individual buffalo (161, 178, and 185) meeting the inclusion criteria described in the methods were used to identify SNV occurring within the mutation spectrum of persistent viruses.

Reads from ancestral 161–1 were assembled to the closest reference genome selected from public databases, i.e. FMDV type O isolate BHU_27/2004 (Accession number HQ268524). The consensus sequences of 161–1 and 161–2 were 100% identical (Table 2). Nine nucleotide variants were detected in 161–2 with frequencies ranging between 27 to 41% (minor variant, Table 3). Variants reported with higher frequency for progeny 161–2 virus corresponded to the nucleotide showing dominance in the sequence of ancestral virus. In all cases the SNVs identified in the progeny virus were also found within quasispecies of the ancestral virus (Table 3). Predicted nucleotide variants that induce non-synonymous amino acid changes were reported [42].

**Table 3:** Nucleotide variants called in sample 161-2 relative to ancestral 161-1 genome 56 d post-sampling—Serotype O.

**Genome position**	**59**		**68**		**1739**		**2870**		**3366**		**3727**		**3863**		**4212**		**6830**	
Corresponding viral protein or structure	5’UTR		5’UTR		VP4		VP3		VP3		VP1		VP1		2B		3D	
SNV detection algorithm^1^	Q		Q		Q		Q		Q		Q		Q		Q		Q	
Ancestral consensus (161–1)	**C**		**T**		**C**		**T**		**T**		**C**		**T**		**A**		**T**	
Alleles in ancestral virus (161–1)	C, G		T, C		C, T		T, C		T, C		C, T		C, T		A, T		T, C	
Alleles in progeny (161–2)^2^	C*	G	C	T*	C*	T	C	T*	C	T*	C*	T	C	T*	A*	T	C	T*
Frequency (%) of alleles in progeny	72	27	34	66	66	33	41	59	30	68	59	41	33	67	50	32	34	66
Count	151	57	155	304	727	369	648	939	300	686	262	179	154	316	594	387	116	224
Coverage	210		459		1110		1587		1016		442		470		1191		340	
Average quality (Phred score)	32	33	33	32	33	33	30	30	33	30	32	33	32	32	32	30	34	32
Non-synonymous amino acid change	-	-	-	-	-	-	-	-	-	-	A880V		-	-	-	-	-	-

^1^Detection method: quality (Q) and/or probabilistic (P).

^2^ Major allele is denoted with an asterisk (*).

Reads from ancestral 178–2 virus were assembled to isolate TUR/13/2013 (Accession number KM268898). At the consensus level sequence 178–2 was 99.9% similar to 178–3 (Table 4). Six SNVs (bolded in Table 4) were detected and denote changes in progeny virus from the dominant allele present in the ancestral genome. Five nucleotide sites (italicized in Table 4) in the progeny virus had minor variants with frequencies between 26 to 48% as compared to consensus 178–2 (Table 4). Three of the predicted SNV would result in non-synonymous aa substitutions (Table 4).

**Table 4:** Nucleotide variants called in sample 178-3 relative to ancestral 178-2 genome 97 d post-sampling—Serotype Asia1.

**Genome position**	**797**		**841**	**1998**	**2543**		**2593**	**2641**	**3238**		**3373**		**3395**		**6082**	**7624**
Corresponding viral protein or structure	5’UTR		5’UTR	VP2	VP2		VP2	VP3	VP3		VP1		VP1		3C	3D
SNV detection algorithm^1^	Q		Q&P	Q&P	Q		Q&P	Q&P	Q		Q		Q		Q&P	Q&P
Ancestral consensus(178–2)	A		A	T	A		A	C	C		C		C		A	A
Alleles in ancestral sequence (178–2)	A		A, C	T, C	A		A, G	C, T	C, T		C		C		A, G	A, G
Alleles in progeny (178–3)^2^	A*	*C*	**C**	C*	A*	*G*	G*	**T**	C*	*T*	C*	*T*	C*	*T*	**G**	G*
Frequency of alleles in progeny	57	43	100	98	52	48	99	100	74	26	58	42	59	41	100	94
Count	193	147	327	586	213	196	391	387	381	134	349	257	352	248	506	374
Coverage	340		327	599	409		393	387	515		606		601		507	400
Average quality (Phred score)	36	37	37	37	37	36	37	37	36	35	37	37	37	37	36	36
Non-synonymous amino acid change	-		-	I302T	K484E		-	-	-		-		P768S		-	-

^1^ Detection method: quality (Q) and/or probabilistic (P).

^2^ Major allele is denoted with an asterisk (*).

The closest reference genome for assembly of 185–1 was Asia-1 isolate TUR/13/2013 (Accession number KM268898). Viruses 185–1 and 185–3 were 99.96% similar at the consensus level (Table 5). In sample 185–3, 4 minor variants were detected with frequencies between 41 to 48%. Only SNV at position 2144 was non-synonymous (H351R). Overall, we observed low inter- and intra-host genomic variability under established persistence of FMDV, with few non-synonymous aa substitutions detected in progeny viruses compared to ancestral ones.

**Table 5:** Nucleotide variants called in progeny 185-3 relative to ancestral 185-1 ancestral virus, 152 d post-sampling—Serotype Asia1.

**Nucleotide position**	**2144**		**2151**		**2794**		**3672**	
Corresponding viral protein or structure	VP2		VP2		VP3		VP1	
SNV detection algorithm^2^	Q&P		Q		Q		Q&P	
Ancestral (185–1) consensus nt	A		T		A		G	
Alleles in ancestral 185–1	A, G		T		A, C		A, G	
Alleles in progeny (185–3)^1^	A	G*	C	T*	A*	C	A*	G
Frequency of alleles in progeny	48	52	47	53	59	41	58	42
Position Count of SNVs in progeny	148	160	136	156	193	133	144	105
Coverage at nt	308		292		329		250	
Average quality in progeny (Phred score)	35	35	36	35	36	36	31	30
Non-synonymous amino acid change	H351R		-		-		-	

^1^ Detection method: quality (Q) and/or probabilistic (P).

^2^ Major variant is denoted with an asterisk (*).

### Evolutionary studies on FMDV persistent viruses evidence trends of purifying and positive selection

The comparison between the number of non-synonymous mutations (dn or Ka), and the number of synonymous mutations (ds or Ks) can suggest whether natural selection is acting to eliminate deleterious mutations (purifying selection) or fix advantageous mutations (positive selection) [33]. To assess the selective pressures exerted on FMDV during persistent infections, we compared the Ka/Ks ratio of FMDV consensus polyprotein sequence derived from the same host at different sampling times (Table 6). The consensus polyprotein sequence was the same in samples 161–1 and 161–2 (Ka/Ks = infinity, Table 6). Non-synonymous mutations were absent (Ka/Ks = 0) in sample 192–3 as compared to 192–2. In all remaining cases, Ka/Ks ratios were below 1, indicative of purifying selection (Table 6). Analysis of Ka/Ks ratios within P1, P2, or P3 regions of the genome also revealed Ka/Ks ratios < 1. The highest Ka/Ks ratio (>0.8) was observed in the P1 region (Table 6).

**Table 6:** Pairwise comparison of Ka/Ks ratio pressure on FMDV polyprotein sequences during persistent infections.

Ancestral sequence	Progeny sequence	Ka/Ks^a^ in polyprotein	Ka/Ks^a^ in P1 region	Ka/Ks^a^ in P2 region	Ka/Ks^a^ in P3 region
161–1	161–2	Ks = 0	Ks = 0	Ks = 0	Ks = 0
192–2	192–3	0	0	0	0
192–2	192–4	0.69	0.41	0	Ks = 0
192–3	192–4	0.46	0.41	Ks = 0	0.33
178–2	178–3	0.23	0.41	Ks = 0	0
184–1	184–2	0.23	0	0.64	0
185–1	185–3	0.69	0.82	0	Ks = 0
277–1	277–4	0.69	0.82	0.32	0.67
254–1	254–4	0.69	0.55	0.64	0.67

^**a**^Calculated with JC = Jukes-Cantor model, K2P = Kimura’s two-parameter methods.

Despite a pattern of purifying selection in samples derived from the same host over an extended time period, four codons may be subjected to positive selection in the alignment of Asia-1 viruses following sequence comparisons made on a phylogenetic rather than pairwise basis (Table 7). Positively selected sites may be useful for identification of antigenic sites involved in viral escape. Three of those predicted codons were located in the structural protein region (N358D, V734T, and T863A) and one in non-structural protein 2B (I1078V). These predictions were supported to variable degrees by FEL, REL, MEME and FADE tests (Table 7).

**Table 7:** Codon sites in the Asia-1 alignment subjected to positive selection.

	FMDV polyprotein regions			
	P1	P1	P1	2B
Codon Substitution	N358D	V734T	T863A	I1078V
FEL (p-value)	ns	ns	ns	0.1
REL Bayes Factor	171.5	175.4	67.8	219.1
MEME (p-value)	ns	0.07	ns	0.06
FADE	1	1	1	1
254–1	HLFDWTPNLAF	ESADPVTSTVE	PEAPRRGDLAA	FGAPILLAGL
8–1	HLFDWTPNLAF	ESADPVTTTVE	QETPRRGDLAA	FGAPILLAGL
137–1	HLFDWTPTLAF	ESADPVTTTVE	QETPRRGDLTA	FGAPILLAGL
277–1	HLFDWTPNLAF	ESADPVTTTVE	QETPRRGDLAA	FGAPILLAGL
185–1	HLFDWTPNLAF	ESADPVTTTVE	QETPRRGDLAA	FGAPILLAGL
184–2	HLFDWTPNLAF	ESADPVTTTVE	QETPRRGDLAA	FGAPILLAGL
178–2	HLFDWTPNLAF	ESADPVTTTVE	QETPRRGDLAA	FGAPILLAGL
192–2	HLFDWTPNLAF	ESADPVTTTVE	QETPRRGDLAA	FGAP**V**LLAGL
192–3	HLFDWTPNLAF	ESADPVTTTVE	QETPRRGDLAA	FGAP**V**LLAGL
178–3	HLFDWTPNLAF	ESADPVTTTVE	QETPRRGDLAA	FGAPILLAGL
185–3	RLFDWTPNLAF	ESADPVTTTVE	QETPRRGDLAA	FGAPILLAGL
189–3	HLFDWTPNLAF	ESADPVTTTVE	QETPRRGDLAA	FGAP**V**LLAGL
192–4	HLFDWTPNLAF	ESADPVTTTAE	QETPRRGDLAA	FGAP**V**LLAGL
277–4	HLFDWTTDLAF	ESADPTTTTVE	QEAPRRGDLAA	FGAPILLAGL
230–4	HLFDWTTDLAF	ESADPTTTTVE	QEAPRRGDLAA	FGAP**V**LLAGL
254–4	HLFDWTTDLAF	ESADPTTTTVE	QEAPRRGDLAA	FGAP**V**LLAGL
231–4	HLFDWTTDLAF	ESADPTTTTVE	QEAPRRGDLAA	FGAP**V**LLAGL

ns: no statistically significant. Underlined letters: Amino acid residues located in P1 subjected to positive selection found in most isolates from last sampling. Bold; Amino acid residues located in 2B subjected to positive selection found in some isolates from second, third and fourth sampling.

### Whole genome and VP1 phylogenetic and recombination analyses of Asia-1 viruses

**Fig 1:**
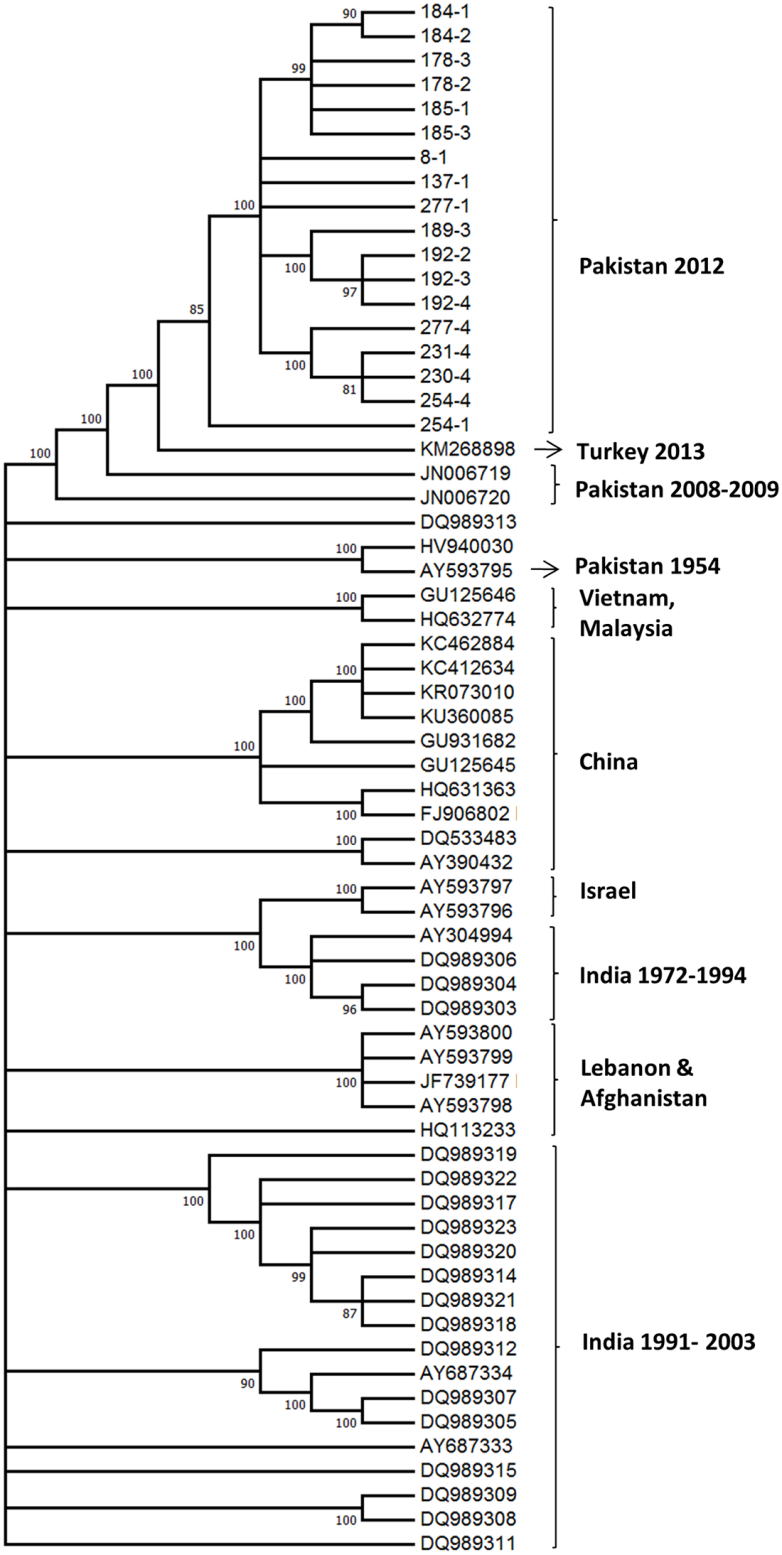
Phylogenetic analysis of Asia-1 FMDV CDS sequences by Maximum Likelihood (ML) method. The evolutionary history inferred by ML method based on complete coding sequence of Asia1 sequences (GTR model). Evolutionary analysis was conducted in MEGA7. The bootstrap consensus tree inferred from 1000 replicates (display threshold = 60%).

To study the genetic relatedness of FMDV Asia-1 viruses from other geographic regions, an ML phylogenetic tree was constructed using genomic sequences of Asia-1 isolates (n = 18) and complete genome sequences available in public databases (n = 46) (Fig 1). All the persistent viruses from buffalo formed a monophyletic clade with high support (bootstrap = 100). Only virus 254–1 grouped as a separate lineage. The closest full-length genomic sequences to those in our study were from viruses from Turkey in 2013 and from Pakistan in 2008–2009. Consistently, those genomes were the closest references used for reads mapping assembly.

**Fig 2:**
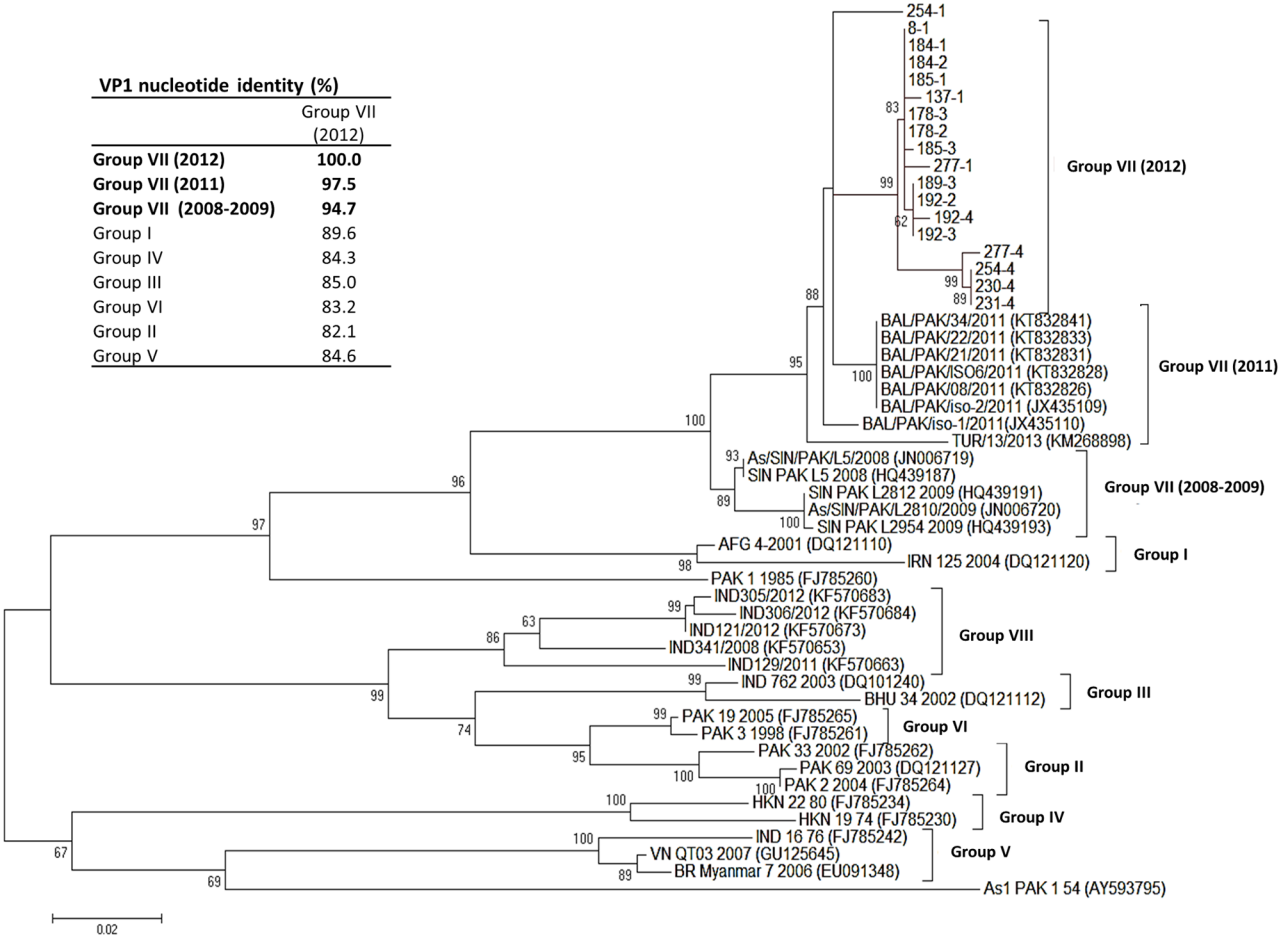
Phylogenetic analysis of Asia-1 FMDV VP1 sequences by ML method. Evolutionary history inferred by ML method for VP1 sequences fragment (size = 633 nt) (HKY model). The tree with the highest log likelihood is shown. The percentage of trees in which the associated taxa clustered together is shown next to the branches. The tree is drawn to scale, with branch lengths measured in the number of substitutions per site. Evolutionary analyses were conducted in MEGA7. I**nset** Percentage of nucleotide identity (%) for groups I-VII as compared to sequences from 2012.

To better understand the phylogenetic relationships of samples from this study in comparison with other Asia-1 isolates, an ML tree was constructed using only the VP1 sequences (Fig 2). The ML tree shows the groups originally proposed by Valarcher, et al. (2009) [31]. The sublineages described by Valarcher, et al. (2009) were defined by 95–100% nucleotide identity across members of each group. When viruses from this study (Pakistan 2011–2012) are compared to the oldest members of group VII (Pakistan 2008 and 2009), nucleotide identity is borderline to justify inclusion in Group VII (94.7%). However, >97% identity is found when isolates of this study are compared to isolates from 2011 [16] derived from cattle clinically suspected for FMD (Fig 2, inset).

**Fig 3:**
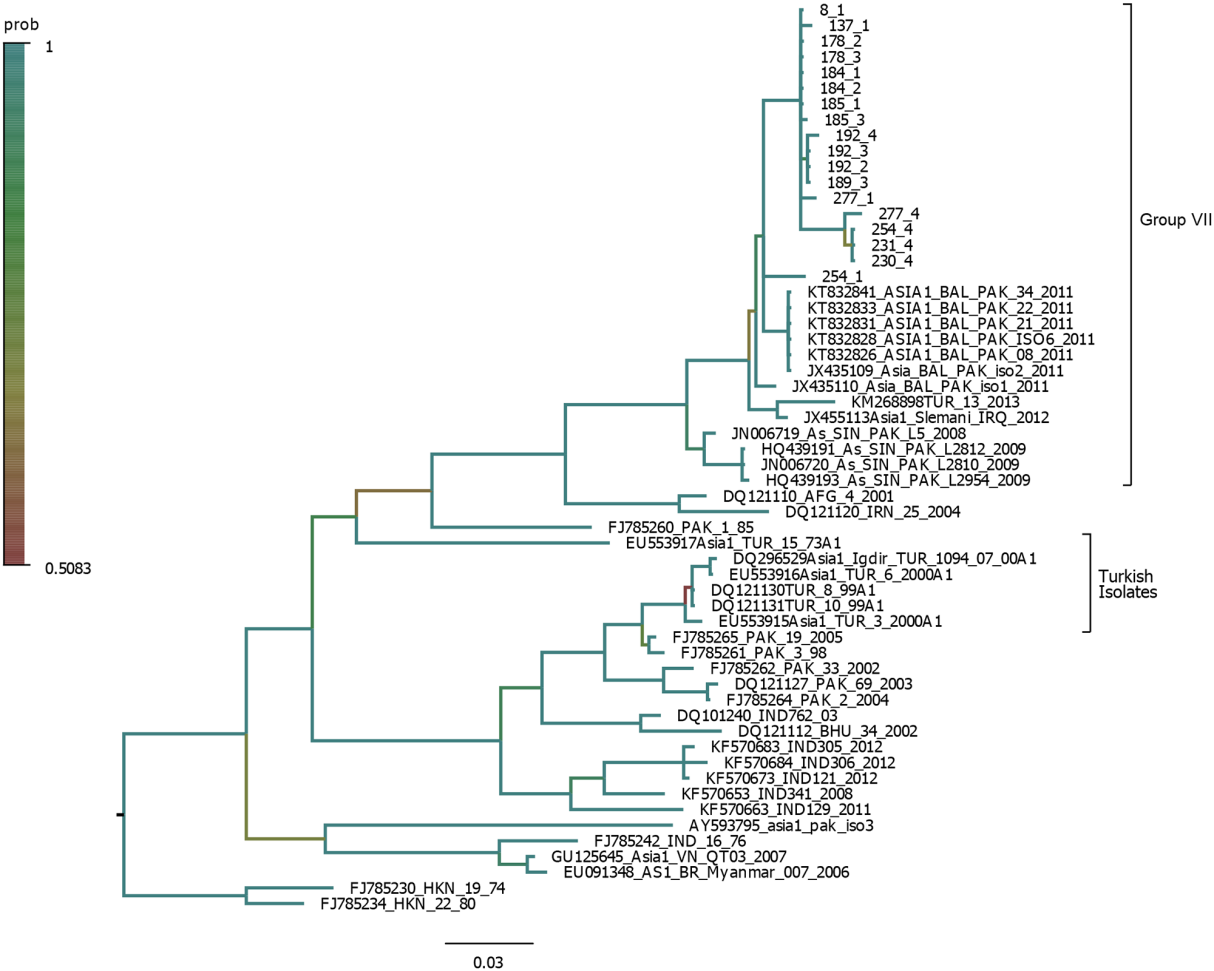
Bayesian phylogenetic analysis of the VP1 nucleotide sequence of Asia-1 isolates from Pakistan and related published regional sequences. Posterior probability values are color coded.

Phylogenetic analysis of VP1 also revealed that most isolates from the fourth (latest) sampling period clustered in a sub-lineage within group VII (2012) but closely related to viruses from sampling periods 1 and 3 (Fig 2). Interestingly, sample 254–1 displayed the lowest nucleotide identity (~97–98%) compared to other Asia-1 isolates from this study and did not group with a virus derived from the same animal in the fourth sampling (254–4) (Figs 2 and 3). Similarly, viral sequences derived from animal 277 from samplings 1 and 4 were divergent and grouped in separate phylogenetic clusters (Fig 2).

An interesting pattern of sequence conservation was observed among viral genomes from the same animal collected at different time points. Very low divergence was observed in samples from animals 184 (sampling #1 and #4), 185 (sampling #1 and #3), 178 (sampling #2 and #3), and 192 (sampling #2, #3, and #4) (See also Table 2).

The finding of two different serotypes in animal 8, collected 141 days apart, is an example of a FMDV reinfection with a different serotype. The first isolate corresponded to serotype Asia-1 and the second virus from the third sampling corresponded to serotype A. The sequence of sample 8–3 shared 96.58% similarity with serotype A SIN/PAK/L4 2008 (Accession number JN006722), the closest genome available in public databases at the time of the analysis. In order to test the possibility of interserotypic recombination as previously reported in Pakistan [14], we conducted a genomic similarity plot analysis of sample 8–3, 8–1, and SIN/PAK/L4 2008 (S1 Fig). The similarity plot did not support the hypothesis that recombination events occurred involving the ancestral Asia-1 (8–1) and a putative parental serotype A genome SIN/PAK/L4 2008) giving rise to the 8–3 genome (S1 Fig). Additionally, no evidence of intraserotypic recombination was found across Asia-1 sequences from this study by a GARD analysis (not shown).

Since Asia-1 persistent viruses in this study clustered closely with an isolate from Turkey (2013), a Bayesian analysis was conducted to determine the phylogenetic relationship of Pakistani and Turkish isolates (Fig 3). VP1 sequences were used instead of complete genomes because more VP1 sequences from Turkey were available in public databases. We found that Turkish isolates from 1999 and 2000 were phylogenetically distant from Pakistani samples in this study (2011–2012) or the Turkish isolate from 2013. However, the lack of Asia-1 Turkish isolate sequences between 2000 and 2012 prevents us from determining whether the Pakistani viruses described here originated in Turkey. The clustering pattern of the VP1 sequences of this study within group VII was similar between the ML (Fig 2) and Bayesian trees (Fig 3).

### Identification of signature amino acids in persistent viruses

**Fig 4:**
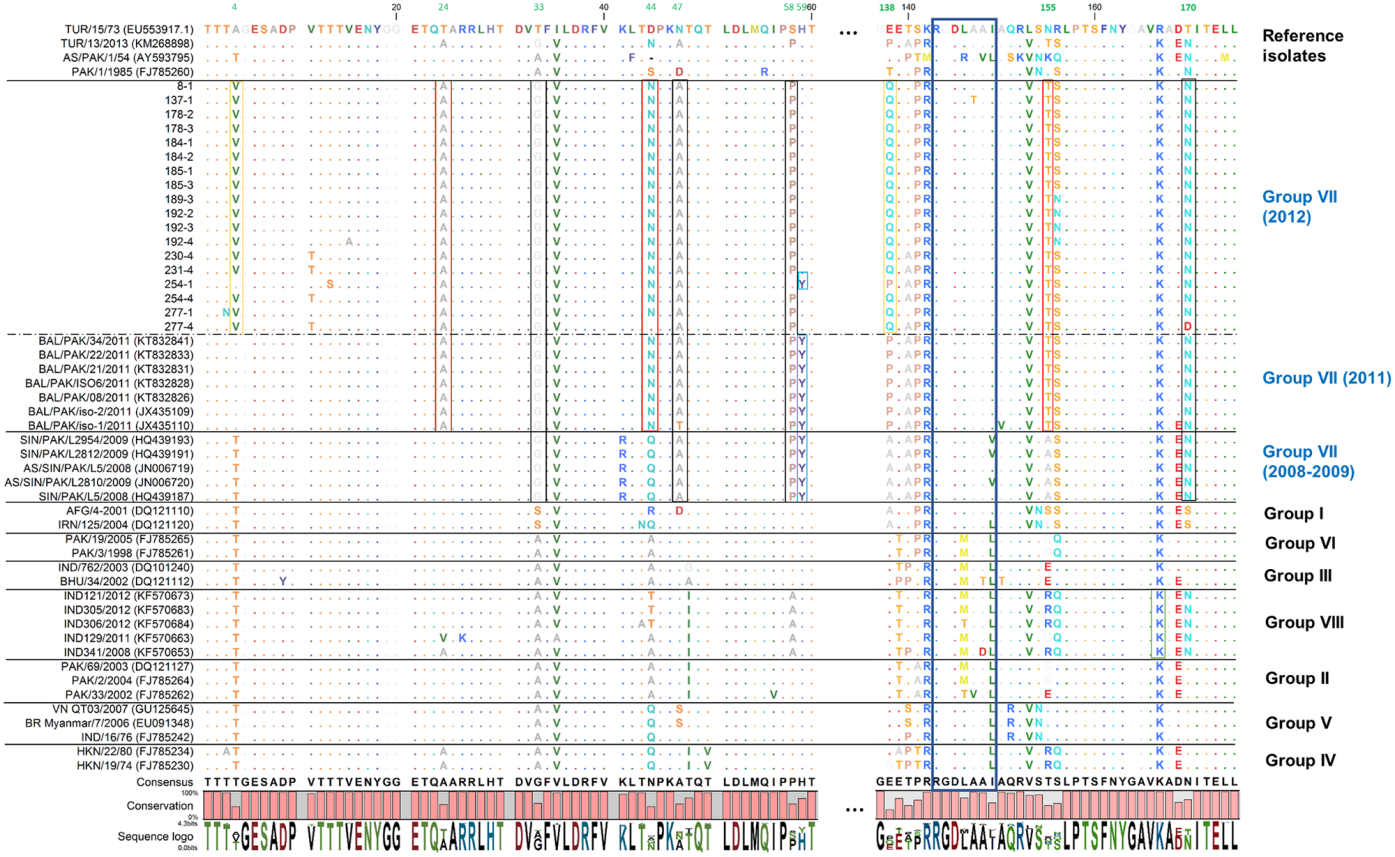
Signature amino acid residues in VP1 of Asia-1 isolates from groups I-VIII. Residues outlined in yellow boxes represent amino acids characteristic from isolates of 2012. Residues outlined in red boxes represent amino acids conserved in isolates from 2011 and 2012. Residues conserved across viruses belonging to group VII are outlined in black. RGD domain is highlighted in a blue box.

Deduced amino acid sequences of VP1 serotype Asia-1 were used to identify signature amino acid changes in persistent viruses from this study as compared to viruses from previously described groups (I-VIII) (Fig 4). We found that residues 4V and Q138 (yellow outlined boxes) were characteristic for samples of this study (Pakistan 2012). The exception was sequence 254–1 which displayed the same residues in those positions as isolates from Pakistan causing outbreaks in 2011. Interestingly 59Y (blue outlined box), described for group VII isolates from 2008 and 2009, was found in isolates from 2011 and sample 254–1 but not in the isolates from 2012, consistent with the hypothesis that virus 254–1 is closely related to sequences from 2011 and ancestral to the remaining isolates from 2012. The aa residues 24A, 44N (except 277–4) and 155T (red outlined box) were found in isolates from 2011 and 2012 but not in viruses from 2008–2009. Residues 33G, 47A (except JX435110), 58P (except 254–1) are found in all members of group VII (black outline boxes) and have been previously described [14]. Residue 140A, described as characteristic for group VII, was found in isolates from 2011 but only in 27% of the persistent viruses in this study (2012). Similarly, residue 170N, originally described as characteristic for group VII, became widespread in groups VIII and all members of group VII (except 277–4) (outlined green box). Other group-specific residues described by Jamal et al (2010) were found in the alignment of representative members of established groups (I-VI) such as 33S in group I and 80I, 86I in group II. The “RGDLXXL” motif within VP1 has been linked to FMDV attachment to the host cell receptor [43]. We found that all isolates belonging to group VII (from 2012 and 2011), except by 137–1, contained the same “RGDLAAI” motif (blue outlined box). In contrast, isolates from 2008–2009 contain both “RGDLAAI” and “RGDLAAV” motifs.

Horsington and Zhang (2007) described the amino acid change (Y→H) in the VP2 protein of persistently infected animals as compared to inoculum virus. In the case of persistent viruses from serotype O described in this study, analysis of VP2 amino acid sequence evidenced the presence of Y at position 79 (not shown). The corresponding nucleotide at position 235 of VP2, did not show any minor variant despite a coverage of 362x (not shown).

### Identification of previously described and novel conserved antigenic sites in Asia-1 isolates

**Fig 5:**
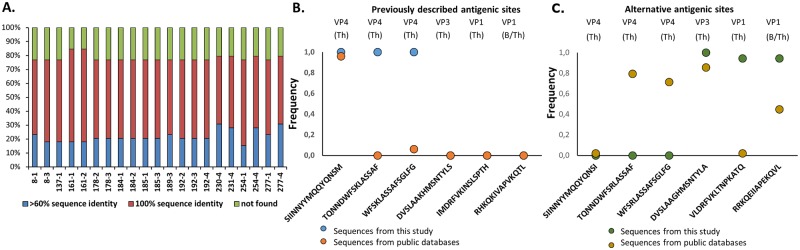
Antigenic sites mapped to deduced amino acid sequences of FMDV isolates. **(A)** Percentage of antigenic sites classified as conserved, alternative and not identified from FMDV persistent isolates from Pakistan (2012) compared to sequences cited by Cooke et al. 2008 **(B)** Sequence and frequency found for conserved antigenic sites located on structural proteins of Asia1 persistent viruses and other sequences available at NCBI database **(C)** Sequence and frequency found for alternative antigenic sites located on structural proteins of Asia1 persistent viruses and other sequences available at NCBI database. In all cases, motifs search was conducted using CLC Genomics Workbench version 8.5.1.

Antigenic variation is a key obstacle for FMD control because it leads to the lack of cross-protection between serotypes and even between strains of the same serotype. To assess the genetic variability at specific antigenic sites, we selected 39 previously described conserved antigenic sites [38] for mapping across the FMDV polyprotein of all 21 isolates derived from persistent infections. Of those, ~half of the epitopes had 100% sequence identity with the sequence of the conserved antigenic site (Fig 5A), ~25% of these motifs were considered alternative epitopes as they displayed >60% of similarity with the described sequence (Fig 5A), and 25% did not map with a sequence similarity >60%.

To identity conserved antigenic sites in structural proteins across Asia-1 sequences, a subset of epitopes of structural proteins (VP1, VP4 and VP3) [38] were searched across 49 Asia-1 polyprotein sequences deposited in Genbank or the isolates from this study. Epitopes SIINNYYMQQYQNSM, TQNNDWFSKLASSAF and WFSKLASSAFSGLFG were conserved across Asia-1 isolates from this study (frequency = 1), but only SIINNYYMQQYQNSM site in VP4 had a frequency > 0.95 across all Asia-1 sequences analyzed (Fig 5B). Two alternative antigenic sites, DVSLAAGHMSNTYLA and RRKQEIIAPEKQTL had a frequency >0.9 across samples from this study. These sites contain few mismatches as compared to previously described antigenic sites (Fig 5C).

## Discussion

This study afforded us the unique opportunity to analyze genetic variation of FMDV during long-term subclinical and persistent infections in Asian buffalo. Our data evidenced low inter- and intra-host genomic variability during persistence of FMDV in Asian buffalo, with overall purifying selection detected. Interestingly, we identified few amino acids within structural proteins subjected to positive selection. We also identified an antigenic motif in VP4 conserved across persistent and non-persistent Asia-1 viruses. Phylogenetic inferences evidenced the relationship of serotype Asia-1 persistent FMDVs reported in this study (2012) to previously reported clinical isolates (2011) members of Group-VII [31], a predominant strain in the West Eurasian area [16]. However, we found evidence of circulation of strains with substitutions in the antigenically relevant VP1 protein within Group-VII (Sindh-08 lineage) in acute [16] and persistently infected animals in 2011. This finding might be relevant to vaccine antigen selection for this region.

NGS techniques create massive amounts of sequence data in parallel [44]. Here, we employed a SISPA approach [23] to multiplex several samples in a single instrument run. The SISPA method produces low sequence coverage at the 3’ and 5’ ends of viral genomes [45] which was evidenced in our data. Heterogeneous depth of coverage across the genome hindered our ability to detect SNVs or to include samples for pairwise analysis in areas with low coverage. Structural elements of FMDV have been reported to constrain NGS sequencing [46] and viral-specific oligonucleotides based on conserved sequences have been suggested to improve sequencing [45]. The SISPA protocol was tested in both platforms (Ion Torrent PGM and MiSeq) to sequence viral genomes [23] but direct comparison of the ability to detect SNVs in each platform was not conducted. It has been described that the MiSeq technology induces lower error rates than the Ion Torrent PGM [47]. However, the Ion Torrent PGM can reach a higher throughput but it also produces homopolymer-associated indel errors [47].

Given the high substitution rate of RNA viruses in relatively short time-scales (i.e. months) [48] the genetic stability observed in this study over several months in persistent infections was unexpected. In most cases, we found >98% nt sequence identity at the consensus level in paired isolates of established persistent infections from the same host (exceptions are samples from animals 254 and 8). In accordance with our findings, a previous study in controlled experimental settings report between 98.9 to 99.7% nucleotide identity between the original inoculation and carrier viruses after up to 98 days post challenge (dpc) [49]. A previous intra- and inter-host diversity study during bovine acute infections reported 13 consensus-level mutations across distinct sample types over the course of 14 days [50].

In our study, intra-host analysis of minor nucleotide variants derived from persistent viruses compared to their ancestral sequence revealed few nucleotide variants (4 to 11 variants per pair of sequences). To avoid false detection of variants we applied stringent filters to account for variation in the depth of coverage and possible systematic base call errors of the NGS technologies. Therefore, our analysis does not provide quantitative information on the total number of variants predicted for a given viral genome. Due to the low quantity of virus in probang samples, it is very difficult to recover enough good quality viral RNA for subsequent NGS library preparation. Therefore, we subjected all the samples to one passage in cell culture. We acknowledge that cell culture passage could introduce some mutations, however, we tried to minimize this by passaging the samples only once in cell culture, unlike other studies where viruses are passaged 2–3 times to make inferences of phylogenetic relatedness and genome diversity [51].

Intra-host analysis of Ka/Ks ratios on viral polyprotein sequences suggests the occurrence of more synonymous changes than non-synonymous changes and evolutionary pressure to conserve the ancestral state—i.e. purifying selection, possibly purging changes that cause deleterious impacts on viral fitness. This could reflect specific adaptation of the virus to the host during persistence. Unfortunately, we cannot compare the genetic divergence and selective pressures exerted on a virus during the transition from acute infection to a persistent state, as our study was carried out under subclinical or already established persistent infections and we did not have access to the original infecting virus. The low nucleotide divergence observed during long-term intra-host infection could be suggestive of low replication rates and/or low selective forces (such as immunological pressure) exerted at the site of persistent infection [i.e. pharynx]. Interestingly, there is evidence of FMDV tropism at sites of persistent infection with favorable conditions for long term survival of infected cells [52] and with reduced immunological pressure [18, 53].

An investigation of serotype O persistently infected cattle reported up to 63 nt changes and 24 aa substitutions in the ORF in persistent infections but none of the aa was consistently found in all the isolates [48]. In serotype O isolates of our study, we found that tyrosine residue 79 of VP2 was a substituted by histidine (Y79H). Previous studies have hypothesized that this substitution may be important for persistence of serotype O viruses, as this residue is located in the B–C loop of VP2 in association with other antigenic sites [54]. However, another study also reports the Y79H substitution in acutely-infected animals [48].

Our analysis of Asia-1 genomes detected few positively selected codons. Four out of five isolates from the fourth sampling displayed positively selected codons in P1, consistent with the clustering pattern observed during the phylogenetic analysis where these isolates formed a monophyletic group. Positive selection has been reported on 1 to 7% of codon positions in FMDV capsid genes for other serotypes in field isolates [55]. More studies are needed to elucidate the mechanisms underlying the FMDV carrier state, including the host-virus interactions that determine dynamics of viral evolution.

Phylogenetic analyses indicated that persistent viruses from this study clustered closely with viruses from acute infections circulating in Pakistan during 2011 [16], and with a single Turkish isolate from 2013 but not with older Turkish isolates (1999–2000). Similar tree topologies were observed when either the full polyprotein or VP1 sequences were used to infer phylogenetic relationships. However, using a larger number of VP1 sequences resulted in better tree resolution. Interestingly, samples 254–1 and 254–4 obtained from the same animal 293 days apart were phylogenetically very divergent (Figs 2 and 3). The observed genetic divergence could be caused by genetic drift or could be the product of reinfection with a different strain of the same serotype. In the case of Influenza viruses, it has been reported that rapid genetic drift can change the antigenic makeup of the virus sufficiently to allow reinfection of patients [56]. Most likely the case of the samples 254–1 and 254–4 represents a reinfection with the same serotype but with a new variant that started circulating in Pakistan in 2011. To our knowledge, reinfection with the same serotype is rare in FMDV and this finding might be the first report of such event.

Different methods have been proposed to classify FMDV strains, subtypes, and sublineages [57, 58]. We and others [14, 16] have followed the grouping system proposed by Valarcher et al. (2009) in which Asia-1 FMDVs from 2003–2007 were divided into six Groups (I-VI). Jamal et al. (2011) defined an additional group VII, later named as Sindh-08 by WRL-FMD [14, 58]. According to this classification, sequences of the persistent viruses from this study were 94–97% similar to viruses within group VII. Group VII is mainly composed by Pakistani viruses (2009–2012) and more recently one virus from Iraq (2012) and an isolate from Turkey (2013).

Antigenic variation is a key obstacle for FMD control; it leads to the lack of cross-protection between serotypes and sometimes between strains [11, 14, 59]. In countries such as Pakistan trying to control FMD using vaccination, field strains that rapidly change present a challenge for the identification of an effective vaccine. Thus, optimal selection of vaccine strains should be addressed through genetic and antigenic characterization of newly circulating viruses [60]. Our phylogenetic studies are consistent with other studies in suggesting that based on their distant phylogenetic relationship, Asia-1 viruses described here might not be adequately covered by the Asia1/Shamir (ISR/89) vaccine [61]. Here we identified signature amino acids, conserved antigenic sites, and the RGD receptor binding motif associated with these viruses. We also identified a T_h_ epitope in VP4 that was conserved across most of the Asia-1 sequences. Knowledge of signature amino acid sequences, receptor binding motifs, and key antigenic sites could be applied to epitope based or chimeric vaccine research [62].

The association between viral genetic sequence and antigenicity is not well defined; consequently, vaccine matching studies using serology are necessary to monitor cross-protection of current vaccines. A better understanding of mechanisms driving virus evolution, particularly understanding selective forces exerted under subclinical and persistent infections might help inform the development of more effective FMD vaccines.

## Supporting information

S1 Fig Genomic similarity plot comparing sample 8–3, 8–1, and SIN/PAK/L4 2008. (TIF)
