# Supplementary figures and images for: Genetic stability of foot-and-mouth disease virus during long-term infections in natural hosts
Source: PLoS One. 2018 Feb 1;13(2):e0190977. doi: 10.1371/journal.pone.0190977 (PMC5794060; doi:10.1371/journal.pone.0190977)

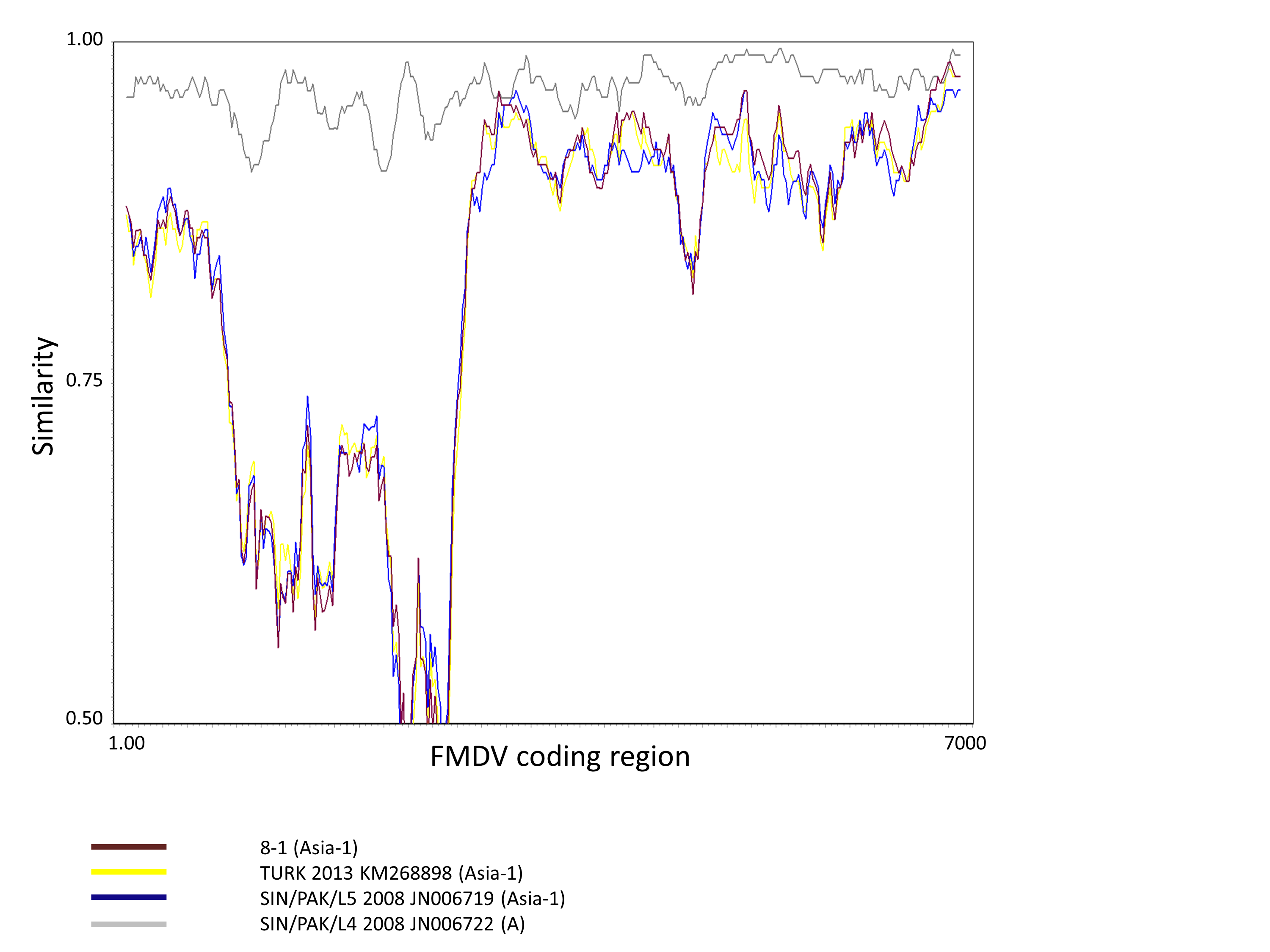

Supplement: S1 Fig — (TIF) [file pone.0190977.s001.tif]
